# The use of emotional intelligence skills in combating burnout among residency and fellowship program directors

**DOI:** 10.1186/s12909-022-03187-z

**Published:** 2022-02-25

**Authors:** Eiman Khesroh, Melissa Butt, Annahieta Kalantari, Douglas L. Leslie, Sarah Bronson, Andrea Rigby, Betsy Aumiller

**Affiliations:** 1 Department of Public Health Sciences, Penn State College of Medicine, Hershey, PA USA; 2 Department of Family and Community Medicine, Penn State College of Medicine, Hershey, PA USA; 3 Department of Emergency Medicine, Penn State Milton S. Hershey Medical Center, Hershey, PA USA; 4 Department of Cellular and Molecular Physiology, Penn State College of Medicine, Hershey, PA USA; 5 Department of Surgery – Division of Minimally Invasive Surgery, Penn State Milton S. Hershey Medical Center, Hershey, PA USA

**Keywords:** Program directors, Physician burnout, Emotional intelligence, Retention, Turnover

## Abstract

**Background:**

Current rates of burnout among physicians are alarming when compared to nonphysician U.S. workers, and numerous interventions have been introduced to mitigate the issue. However, no interventions have specifically targeted the 30% burnout rate among physician program directors. The complex and demanding role of program directors necessitates building relationships, solving crises, securing jobs for residents and maintaining well-being of trainees. The aim of this study is to investigate the impact of emotional intelligence (EQ) on burnout levels among program directors.

**Methods:**

A cross-sectional survey was administered from May 17 to June 30, 2021 to program directors and assistant/ associate program directors at an academic medical center in south-central Pennsylvania. A self-report questionnaire was used to collect data. The survey included an open-ended question along with the Trait Emotional Intelligence Questionnaire- Short Form (TEIQue-SF), Copenhagen Burnout Inventory (CBI), and demographic questions. All data were analyzed using SAS Version 9.4.

**Results:**

Of the 109 program directors and assistant/associate program directors invited in the survey, 34 (31.20%) responded. The findings indicate that there is a moderate inverse association between EQ and burnout, suggesting EQ as a protective factor against burnout. We also found that program directors who were considering leaving their position demonstrated higher levels of burnout compared to those who did not. Results from the open-ended question suggest that perceived lack of support, micromanagement, criticism, and extra duties with less payment were among the reasons program directors and associates were considering steeping down from their position. The results showed no association between EQ skills and years of practicing.

**Conclusions:**

Burnout among program directors and assistant/associate program directors is not as alarming as rates of burnout among physicians-in-training. However, despite high level of EQ skills and low burnout level, nearly 43% of program directors were considering leaving their position. Nurturing EQ skills may be useful in improving retention and reducing turnover among medical leaders.

## Background

The term “burnout”, which is defined by the World Health Organization (WHO) as a long-standing work-related stress [1], is becoming well recognized in the medical field, with incidence rates exceeding those of nonphysician U.S. workers (48.8% vs. 28.4% in 2014) [2]. Several reports have demonstrated the adverse impact of burnout on physicians’ well-being, as well as the risks of increased medical errors (10%), suicide ideation (6.5%), alcohol and substance use (10%), turnover rate (28%), and reduced physician professional effort (50%) [3–6]. Additionally, the literature documented on the adverse consequences of burnout include diminished cognitive functions such as impaired attention, memory and executive functions [7, 8]. Mitigation interventions to reduce burnout have been implemented at both the personal and institutional levels: personal interventions have focused on resilience and mindfulness, while institutional interventions have targeted reducing work hours and overall workloads.

An additional example of an area for personal intervention is emotional intelligence (EQ), which has been defined as the ability to monitor, understand, and guide one’s own thinking and actions [9]. EQ skills such as emotional self-awareness, self-control, adaptability, empathy, teamwork, leadership, and social awareness have been cited as essential constructs for individual professional development and decision-making [9, 10]. Studies have also reported that equipping individuals in a leadership position with EQ skills has had a significant impact, which may not be limited to leader’s well-being and retention, but can also improve the entire team’s well-being and productivity [11–13]. Consequently, EQ skills are becoming a top priority for institutions seeking maximum productivity and efficiency [11].

The association between burnout and EQ has been studied previously, with most research affirming an inverse association between the two, i.e., higher EQ skills correlate with lower levels of burnout [14, 15]. Despite this, EQ has not been considered a vital component in the medical training of physicians. Burnout is also under-researched among residency and fellowship program directors. A recent study documented that burnout levels in internal medicine program directors reached 29% and was associated with pronounced levels of turnover as well as created a negative impact on the continuation of teaching and care for residents and patients [16]. Additionally, the authors reported that factors contributing to burnout among physicians in leadership positions included perceived lack of support from department chairs and hospital administrators, lack of training in the leadership role, and poor relationships with colleagues [16]. These contributing factors suggest that it could be helpful to enhance EQ skills among leaders. The aim of the present study is to investigate the impact of program directors’ EQ skills on burnout level. We hypothesize that program directors with higher EQ skills experience lower levels of burnout.

## Methods

### Aim, design, setting

The primary objective of the study is to evaluate the association between EQ skills of residency and fellowship program directors, including assistant/associate program directors, and level of burnout. A cross-sectional survey including fellowship and residency program directors and assistant/associate program directors at an academic medical center in south-central Pennsylvania was conducted from May 17, 2021, through June 30, 2021. Recruitment involved obtaining a list of fellowship and residency program directors and assistant/associate program directors from the director of graduate medical education at the institution and contacting the prospective participants via email. Program leaders were sent a link containing the study’s consent material; if they agreed to participate, they were taken to the survey. The survey was administered through REDCap, a secure online web application [17].

#### Materials

The survey included two instruments: the Copenhagen Burnout Inventory (CBI) and Trait Emotional Intelligence Questionnaire-Short Form (TEIQue-SF). The CBI is a publicly available and validated burnout instrument with high internal reliability and consists of 19 questions divided into three subcategories: personal-, work-, and colleague-related burnout. Each category is evaluated on two physical and psychological cores: exhaustion and fatigue [18]. Participants responded to each question using a 5-point Likert scale ranging from 1 (always) to 5 (never), with aggregate scores ranging from 0 to 100. Results were trisected into low, medium, and high scores as per instrument guidelines [19].

The TEIQue-SF is a 30-item validated instrument [20] that provides a global assessment of four scales: well-being (WB), self-control (SC), sociability (SOC), and emotionality (EM) [20, 21]. The overall assessment of the WB domain provides an insight into how happy and positive an individual is; the SC domain determines the extent to which an individual is able to cope with external stressors; SOC examines the individual’s ability to listen and communicate in a self-assertive manner; and EM evaluates the individual’s ability to express and interpret emotions needed to establish and maintain a relationship with others. The TEIQue-SF is scored on a 7-point Likert scale ranging from 1 (completely disagree) to 7 (completely agree). In addition to the CBI and TEIQue-SF instruments, the survey included a demographic section where participants provided information regarding their age, gender, and marital status, as well as whether program directors ever considered leaving their position and the leading reasons behind this decision. For those who responded that they did have a desire to leave the institution, an additional open-ended question was posed inquiring about if they ever wanted to leave their position as program directors.

A number of studies have highlighted the additional strain that the COVID-19 pandemic placed on medical education at all levels [22, 23]. As this research took place during the COVID-19 pandemic, a single item to measure the leaders’ perceived stress resulting from the impact of the COVID-19 pandemic was included in the questionnaire. This item asked the program director to rate their level of agreement that the COVID-19 pandemic adversely impacted their emotional exhaustion level with “1″ being strongly agree and “5″ being strongly disagree.

#### Statistical analysis

Descriptive statistics were used to characterize the study sample. Means and standard deviations (SD) were used to analyze continuous variables. Frequencies and percentages were used to present any categorical variables. The association between two continuous variables was evaluated using the Pearson Correlation Coefficient (PCC) and 95% Confidence Limits (CL) and linear regression models. Linear associations between EQ and burnout skills were controlled for by the perceived impact of the COVID-19 pandemic. Associations between continuous and categorical variables were calculated using Spearman Correlation Coefficients (SCC) and 95%CL along with ANOVA. All data were analyzed using SAS version 9.4 (SAS Institute Inc., Cary, NC).

## Results

Of the 109 program leaders (directors and associates) invited to the study, 34 (31.2%) elected to participate. Table 1 shows that, out of the 34 respondents, 20 (58.82%) were male, and more than half of respondents identified as White (*n* = 27; 79.41%). The majority of the sample (*n* = 26; 76.47%) were age 40 or older and were married (*n* = 31; 91.18%). Sixteen respondents (47.06%) reported having been a program director for less than 5 years, while only 8 (23.53%) had a 10-year experience as a program director. The remaining participants (*n* = 10; 29.41%) had been program leaders for 5 to 10 years. Program leaders demonstrated variable levels of burnout, with 22 respondents (68.75%) in the low range of burnout and 10 (31.26%) in the moderate to highest range.

**Table 1:** Demographics (*N* = 34)

	N (%)
Gender	
Male	20 (58.82%)
Female	14 (41.18%)
**Age**	
< 40 years	8 (23.53%)
40+ years	26 (76.47%)
**Race**	
White/ Caucasian	27 (79.41%)
Asian/ Pacific Islander (e.g. Native Hawaiian)	2 (5.88%)
Two or more	2 (5.88%)
Decline to answer	3 (8.82%)
**Marital Status**	
Single/ Widowed	2 (5.88%)
Married/ Significant Other	32 (91.18%)
**Tenure Years**	
< 5 years	16 (47.06%)
5–10 years	10 (29.41%)
> 10 years	8 (23.53%)
**Intentions to Leave Leadership position**	
Yes	15 (42.86%)
No	20 (57.14%)

Table 2 shows that work-related (mean = 42.97; SD = 18.89) burnout was higher than personal- and colleague-related burnout (mean = 40.95; SD = 22.04 and mean = 27.08; SD = 21.96, respectively). Table 2 also shows that program leaders also exhibited a high level of EQ skills across the four subdomains (WB, SC, EM, and SOC), with no relation to years of experience. The PCC (95% CL) showed a moderate inverse association between burnout and EQ (− 0.51 [− 0.73, − 0.16]), which suggests EQ may be a protective factor against burnout. These findings suggest that acquiring high EQ skills could result in lower levels of burnout.

**Table 2:** Emotional Intelligence and Burnout Scores among Program Directors

	Program Directors Mean, SD
EQ Domains	
Well-Being	4.70, 0.92
Self-Control	4.98, 0.91
Emotionality	5.30, 0.84
Sociability	4.72, 1.03
Total	5.19, 0.85
CBI Domains	
Personal	40.95, 18.97
Work	42.97, 18.89
Colleague	27.08, 21.96
Total	38.17, 18.97

**Fig. 1:**
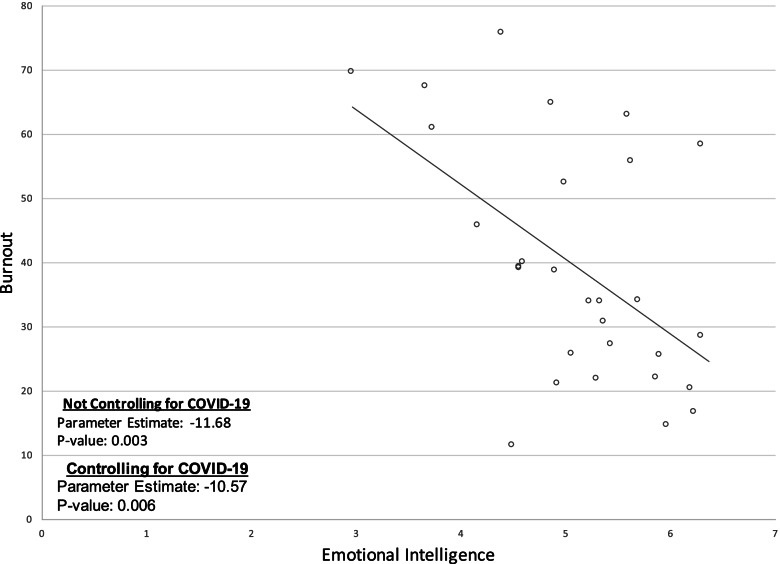
Association of Emotional Intelligence and Burnout Among Training Program Directors

In Table 3, we present associations between burnout and years of tenure, desire to leave position, and impact of COVID-19. Burnout levels remained constant along years of medical practice with a SCC (95% CL) of − 0.05 (− 0.40, 0.31), suggesting no association between burnout level and the number of years a program director has been practicing medicine (*p*-value = 0.94). However, program leaders who indicated an intention to leave their position (*n* = 15; 42.86%) reported a significantly higher levels of burnout compared to those with no intention of leaving (*p*-value = 0.0003). We also looked at the impact of the COVID-19 pandemic on program leaders and found a positive association between COVID-19 and burnout with an SCC (95% CL) of 0.29 (− 0.07, 0.58). Program directors who reported being impacted less by COVID-19 had noticeably lower burnout scores; however, this association was trending towards significance (*p* = 0.09). Figure 1 illustrates the association between EQ and burnout for program leaders and demonstrates that as EQ scores increase, burnout scores decrease (*p* = 0.003). After controlling for the impact of the COVID-19 pandemic, this association remains significant (*p* = 0.006).

**Table 3:** Associations between burnout and years of tenure, desire to leave position, and impact of COVID-19

Variable	Level	N(%)	Mean (SD)	ANOVA *P*-Value	Spearman Correlation Coefficient (95% CL)
Years of Tenure	< 5 years	15 (48.34%)	39.18 (19.08)	0.94	−0.06 [− 0.40, 0.31]
	5–10 years	9 (29.03%)	37.30 (19.74)		
	> 10 years	7 (22.58%)	36.03 (21.71)		
Considered Leaving	No	17 (53.13%)	27.58 (15.78)	0.0003	0.66 [0.39, 0.82]
	Yes	15 (46.86%)	50.17 (14.88)		
Emotionally Exhausted by COVID-19	Agree/Neutral	28 (87.50%)	40.31 (19.15)	0.09	0.29 [−0.07, 0.58]
	Disagree	4 (12.50%)	23.21 (8.64)

An open-ended question was posed to the 15 respondents who stated an intention to leave; of these, 13 indicated that lack of support and micromanagement were the main reasons they were considering a job change. Approximately 15 respondents reported an intention of leaving and 6 (46.15%) out of the 13 respondents who provided a response to the open-ended question indicated that lack of support and micromanagement were the main reasons to considering leaving the position.

## Discussion

The association between burnout and EQ among residents has been frequently investigated [14, 15]. However, to date there has been little research on burnout levels among program directors. The hypothesis of this study—that possession of high EQ skills is associated with lower levels of burnout—was supported by our data. Physician leaders such as residency and fellowship program directors have responsibilities for program administration and operations; recruitment and selection of residents; education; supervision; assessment, promotion, and disciplinary actions; and for establishing an effective learning climate at all teaching sites. Therefore, directors play an essential role in the department and organization [16, 24]. The complexity of program directors’ roles assigned by the Accreditation Council for Graduate Medical Education (ACGME) engendered a burnout level close to 30%, in addition to high turnover rates [16].

The role-specific challenges have been found to be related to frustration with regularity requirements, lack of appreciation and support from the department chair and hospital administration, low satisfaction with colleague relationships, and lack of training in dealing with residents’ problems [16, 25]. These themes related to lack of support and interpersonal challenges were affirmed by some of the respondents in our study; they stated that *“micromanagement from direct boss (department chair), conflicting personalities with select colleagues, opinion not respected, generational differences [between] self and learners, little room for additional growth in this role, not enough time given to focus on program/department/institutional issues related to GME”* and *“criticism of those around me”* were contributing factors to burnout. These sentiments regarding lack of professional support in addition to interpersonal challenges presented in a number of open-ended responses from program leaders and highlight the impact that these particular factors have on the work-related burnout that these leaders experience.

Additionally, the recent abolishment of the protected time for core faculty has left a significant number of physicians, notably assistant program directors who are engaged in medical education and research, to feel unrewarded as one respondent stated that having *“more work and no increase in pay”* to be emotionally draining [26]. These factors suggest the need for further investigation by academic institutions, as well as the ACGME. Physician-leaders in our study who were considering leaving their position cited micromanagement by superiors, lack of departmental and institutional support, and continuous criticism. Some said that burnout stems in part from feeling *“fatigued, less invested in program” and “challenges of always innovating,”* which is consistent with previous research [25].

Being skillful with EQ prepares individuals in leadership positions to delegate, manage conflicts, and build relationships. However, despite the reported high emotional intelligence scores in this study across the four subdomains (WB, SC, SOC, and EM), the data suggest a discrepancy in communication and misalignment in relationships with administrators, physicians-in-training, and colleagues in terms of sharing opinions, respecting one another, and dealing with opposite personalities. To excel at the use of EQ skills, one should be adaptive to using these skills intra- and interpersonally, i.e., helping oneself and others, respectively [27]. One of the four building blocks of EQ demands that the individual has “the ability to accurately perceive your own and others’ emotions; to understand the signals that emotions send about relationships; and to manage your own and others’ emotions” [28]. However, when an individual is burned out or emotionally exhausted, the emotionally guided behaviors and thinking become distorted [27]. Thought distortion was noted in several studies that investigated the influence burnout has on cognitive and executive functions [7, 8]. Memory, attention, flexibility in switching tasks and hence professional performance were found to be impacted [7]. The result is a cycle of unresolved problems among individuals lacking EQ skills.

Program directors are in a position of leading teams, building relationships, influencing careers, and maintaining the well-being of others. They are expected to overcome crises, motivate different personalities, and achieve effective communication for optimal outcomes among the learners. In order to ensure that these responsibilities are delivered suitably while securing program directors’ well-being, the ACGME in addition to the institution should be providing solutions to the factors leading program directors to feel emotionally exhausted. The solution begins with ensuring a positive emotional culture at the institutional level that provides healthy support and appreciation. In addition, if improving the EQ skills at the personal level is found promising to boost the well-being and productivity of program directors, then EQ skills should be administered to all healthcare providers working in that institution to ensure successful interpersonal skills. The role of program directors should not lead to feeling a need *“to pursue other endeavors”* or *“have thought about leaving my job in general*,” as reflected by some respondents, when the solution resides in including program directors in the process of building institutional cultural and emotional competencies.

There are some limitations to this study. First, the sample size is small. Consequently, these results may be impacted by response bias. We tried to increase the number of participants by inviting program directors from all departments as well as assistant and associate programs; this could have influenced the results, as some departments practice differently than others. Additionally, the study was conducted during the beginning of the summer and during the COVID-19 pandemic, when several directors were on leave and others were busy finalizing academic requirements for physicians-in-training preparing to graduate or joining the program.

## Conclusions

High EQ skills are associated with low burnout rates. The positive impact of EQ skills is not only limited to elevating leaders’ well-being and increasing retention; but also improves the well-being and productivity of the team, and contributes to maintaining effective communication and a supportive culture. Regular EQ skills sessions should be included in the process of preparing program directors for their roles. This study calls for further EQ investigation using qualitative methods. It is also a call for the ACGME to investigate the responsibilities assigned to residency and fellowship program directors that could be contributing to experience burnout. Further, given the undeniable impact that the COVID-19 pandemic has had on medical educational [cite] and, by extension, program leaders. Future research should aim to evaluate the impact that the changes that occurred due to the COVID-19 pandemic may have on these associations moving forward.

## Data Availability

The datasets generated and/or analyzed during the current study are not publicly available due to ethical and regulatory restrictions.

## Abbreviations

ACGME: Accreditation Council for Graduate Medical Education
CBI: Copenhagen Burnout Inventory
CL: Confidence Limits
EM: Emotionality
EQ: Emotional intelligence
IRB: Institutional Review Board
N: Number
PCC: Pearson Correlation Coefficient
SC: Self-control
SCC: Spearman Correlation Coefficients
SD: Standard Deviation
SOC: Sociability
TEIQue-SF: Trait Emotional Intelligence Questionnaire – Short Form
WB: Well-being
WHO: World Health Organization
